# Comparison of Oxytetracycline and Sulfamethazine Effects Over Root Elongation in Selected Wild and Crop Plants Commonly Present in the Mediterranean Cropland and Pasture Scenarios

**DOI:** 10.1007/s00244-024-01104-7

**Published:** 2024-12-07

**Authors:** Carlos Sacristán, Marta Guerrero, Sonia Sánchez, Antonio Rodríguez, Rosa María García, Ana Carolina Ewbank, Meritxell Gros, Sara Rodríguez-Mozaz, Isaura Martín Martínez, Luis Guasch, Ana de la Torre

**Affiliations:** 1 Group of Epidemiology and Environmental Health, Animal Health Research Centre (INIA-CISA/CSIC), Carretera Algete-El Casar de Talamanca, Km. 8,1, 28130 Valdeomos, Madrid, Spain; 2 Spanish Plant Genetic Resources Centre (CRF) (INIA-CRF/CSIC), 28805 Alcalá de Henares, Madrid, Spain; 3 INRAE, URP3F, 86600 Lusignan, France; 4 Catalan Institute for Water Research (ICRA), C/ Emili Grahit 101, 17003 Girona, Spain; 5 Universitat de Girona (UdG), 17004 Girona, Spain

## Abstract

Fertilization with animal manure and sewage sludge, and the use of sewage water for irrigation, can lead to high antimicrobial concentrations in agricultural soils. Once in soil, antimicrobials can exert direct and indirect toxic effects on plants by misbalancing plant–microbe symbiotic relationships. We performed germination tests to determine the optimum germination conditions of 24 plant species (10 crop and 14 wild species). Subsequently, we analyzed the differences in oxytetracycline and sulfamethazine phytotoxicity in 19 plant species for which optimum germination conditions could be established. The root elongation of the majority of wild species was inhibited in the presence of oxytetracycline and sulfamethazine, whereas crops were mainly affected by oxytetracycline. There were no differences in sensitivity to oxytetracycline between crop and wild plant species, whereas wild plants were significantly more susceptible to sulfamethazine than crop species. Thus, to cover both productivity and biodiversity protection goals, we recommend pharmaceuticals’ predicted no-effect concentration (PNEC) values based on crop and wild plant species phytotoxicity data.

**Supplementary Information:**

The online version contains supplementary material available at 10.1007/s00244-024-01104-7.

Plant biodiversity plays a critical role in biogeochemical cycles by providing, regulating and supporting a wide range of ecosystem services (Arts et al. 2015; Boutin et al. 2014). In pasture ecosystems, the grassland vegetative cover prevents soil erosion, favors recharge groundwater, and manage floods through increased infiltration and decreased water runoff (Altieri et al. 1999). In pasture and cropland ecosystems, vegetal resources support pollinator services (Hendrickson and Sanderson 2017; Kaluza et al. 2017), and play a role in nutrient recycling and soil conservation by activating soil biota (Altieri et al. 1999). Industrial agriculture usually reduces non-crop plant biodiversity, as it prioritizes productivity of food, raw materials (e.g., fiber, fuel), and incomes over biodiversity (Ortiz et al. 2021). In view of this scenario, current European Union (EU) agricultural policies specifically target the conservation of the agricultural land biodiversity (Hermoso et al. 2022).

Antibiotics are considered emerging pollutants (Singh et al. 2021). Because most antibiotics are not fully metabolized by humans and animals, a significant proportion (up to ~ 90%) is excreted unchanged through feces and urine (Conde Cid et al. 2020). Thus, these compounds can reach the soil through fertilization with animal manure (Ghirardini et al. 2020) and sewage sludge from wastewater treatment plants, and the use of sewage water for irrigation (Vuaille et al. 2022). Of note, animal manure is directly applied to arable crops of cereals (mainly wheat [*Triticum* spp.], barley [*Hordeum vulgare*] and maize [*Zea mays*]), oilseed and protein crops, and to lands used for silage and animal grazing (García et al. 2022). As a result, according to the compound and their sorption potential, antibiotics can reach maximum concentrations (between 4 × 10^–7^ and 50 mg/kg) (Cycon et al. 2019; Conde Cid et al., 2020), and some of them may persist for several years in agricultural soils (Förster et al. 2009). In the EU, according to current regulations, sewage sludge is processed prior to its use as soil amendment (European Council Directive 86/278/EEC). The application of different biological, chemical or thermal treatments significantly reduces the sludge antibiotic content, thus reducing the entry of such pollutants into soil (Verlicchi and Zambello 2015). At concentrations typically found in agricultural soils, antibiotics can exert direct toxic effects on plants by decreasing the germination percentages, plant biomass, branching patterns, shoot and root length, fresh/dry weight, number of leaves, respiration rate or chlorophyll synthesis (Bártíková et al. 2016; Buta et al. 2021; Carballo et al. 2022; Vuaille et al. 2022). Additionally, by misbalancing plant–microbe symbiotic relationships (e.g., plant nutrition, growth and resilience to overcome biotic or abiotic stress promoted by rhizobia bacteria and mycorrhizal fungi), antibiotics also have an indirect effect (Narciso et al. 2023). Although the effects of antibiotics over crop plant species have been investigated, our knowledge regarding wild plants’ potential responses to these compounds in soil is very limited (Carballo et al. 2022). Carballo et al. (2022) performed a critical review to examine the state of knowledge on the phytotoxic effects of antibiotics on terrestrial crop and wild (non-crop) plants of pastureland and crop field margins to evaluate differences in their sensitivity. According to their results, the available dataset was small: 275 datasets for germination and 169 datasets for plant growth (elongation and biomass). Additionally, less than 1% of the data came from wild species, preventing an answer regarding the hypothetical sensitivity differences between wild and crop plant species (Carballo et al. 2022). This gap of knowledge is significant, because the biodiversity protection goal for plants is not foreseen by the current European legislation on pharmaceuticals phytotoxicity. Specifically, the Environmental Risk Assessment (ERA) of veterinary pharmaceuticals is focused on crop productivity, and their phytotoxicity has only been assayed in crop plant species (EMA 2017). There are no provisions for ERA regarding human pharmaceuticals (de la Casa-Resino et al. 2021).

The direct phytotoxicity of antibiotics on plant growth can be estimated by using the early germination test, where seeds are grown on a doped filter paper (ISTA 2016). The main advantages of this test are its fast toxicity results and guarantee of a homogenous substrate for all treatments, preventing variations in soil-related factors (e.g., water holding capacity), and allowing results’ comparison regarding different contaminants and plant species under standardized conditions. One limitation is that although they are good indicators of the general toxicity and magnitude of the toxicity range, it is not possible to directly transfer such results to soil systems (Liu et al. 2009; Timmerer et al. 2020).

In this context, we hypothesized that there are differences in antibiotic phytotoxicity in wild versus crop plants. Thus, the goal of this study was to evaluate if (i) sentinel Mediterranean wild plants are more sensitive than sentinel crop plants to frequently used antibiotics (i.e., oxytetracycline and sulfamethazine), and (ii) the effects of their toxicity, by assessing and comparing root elongation in wild and crop plant species.

## Material and Methods

### Experimental Materials

The antibiotics used in the study were oxytetracycline hydrochloride (class tetracyclines; CAS No 2058–46-0; VETRANAL, analytical standard, Sigma-Aldrich purity > 90.0%, Sigma-Aldrich Steinheim, Germany) and sulfamethazine (class sulfonamides; CAS No 57–68-1; VETRANAL, analytical standard, purity > 98.0%, Sigma-Aldrich). We selected these pharmaceuticals because tetracyclines are the most widely prescribed antibiotics in human medicine (ECDC 2023), and alongside sulfonamides, are among the main veterinary antibiotics sold worlwide (25.8% and 9.9%, respectively) (EMA, 2022). The main characteristics of each antibiotic are shown in Supplementary Table 1.

Crop and wild plant species were selected among those families commonly present in the Mediterranean cropland and pasture scenarios (Garcia et al. 2022). The selected crop plants comprise 10 species of four families, which account for 67% of the Mediterranean’s surface dedicated to agricultural production, i.e., Poaceae (n = 4), Asteraceae (n = 2), Fabaceae (n = 2), and Brassicaceae (n = 2) (Supplementary Table 2). The list of wild plants included eight species, classified into families: Poaceae (n = 3), Asteraceae (n = 2), Fabaceae (n = 2), and Brassicaceae (n = 1). We also included six wild species from other frequent families, in order to reflect wild biodiversity: Hypericaceae (n = 1), Papaveraceae (n = 1), Plantaginaceae (n = 1), Polygonaceae (n = 1), Primulaceae (n = 1), and Rubiaceae (n = 1) (Supplementary Table 2). All of them are listed in the OECD 208 (2006) guidelines. Seeds of the crop and wild plant species selected for this study were obtained from commercial suppliers, and private and public collections (Supplementary Table 2).

### Germination Conditions

Germination tests at different temperature regimes, and thermal, chemical and mechanical treatments were performed to select the appropriate conditions for seed germination. Germination was determined by radicle elongation of at least 2 mm. Tests were performed using the filter paper method, according to the International Seed Testing Association (ISTA) protocols (ISTA 2016).

Seeds were rinsed with distilled water prior to germination tests. All treatments consisted of 2–4 replicates with 100 seeds (25 or 50 seeds in each replicate). The seeds were placed on Filter Lab 516G (85 mm in diameter) and 518G (130 mm in diameter) in disposable plastic Petri dishes (90 and 150 mm). The filter paper was moistened with distilled water (6–14 mL) and sealed with Parafilm, until completion of the experiment (maximum 21 days). Specifically, seeds weighing less than 0.1 g/100 seeds germinated in two 90 mm Petri dishes and moistened with 6 mL of distilled water (i.e., scarlet pimpernel *Anagalis arvensis,* English daisy *Bellis perennis,* common foxglove *Digitalis purpurea*, lettuce *Lactuca sativa,* and timothy grass *Phleum pratense*). Seeds weighing between 0.1-2 g/100 seeds germinated in two 90 mm Petri dishes and moistened with 7 mL distilled water (i.e., rapeseed *Brassica napus,* cabbage *Brassica oleracea,* bittercress *Cardamine pratensis*, cornflower *Centaurea cyanus*, birdsfoot trefoil *Lotus corniculatus*, alfalfa *Medicago sativa,* curled dock *Rumex crispus,* and red clover *Trifolium pratense*). Finally, seeds weighing over 2 g/100 seeds either germinated in two 150 mm Petri dishes and were moistened with 14 mL distilled water (i.e., barley *Hordeum vulgare* and wheat *Triticum aestivum*) or in four 150 mm Petri dishes and moistened with 14 mL (i.e., oat *Avena sativa* and sterile oat *Avena sterilis),* 17 mL (i.e., sunflower *Helianthus annuus* and common vetch *Vicia sativa*), or 21 mL (corn *Zea mays*) distilled water, depending on seed size. Additionally, we worked with four wild plant species—*Echinochloa crusgalli, Hypericum perforatum, Papaver rhoeas,* and *Galium aparine*—that required additional chemical (e.g., bleach disinfection, immersion in sulfuric acid) and mechanical seed treatments (e.g., scalpel blade incision to promote imbibition, use of sea sand to promote superficial seed erosion by agitation; superficial seed erosion by welding) to germinate. Unfortunately, such treatments were not successful in promoting seed germination and, therefore, these species were not included in the subsequent antibiotic assays.

Germination percentage and days until germination were evaluated at different temperature conditions (15 ºC, 20 ºC, 25 ºC, 20/30 ºC), under a 16:8 h light cycle and 95% relative humidity. When germination percentages showed values lower than 70%, additional thermal (i), chemical (ii) or mechanical (iii) treatments were applied: (i) stratification treatment during 14 days prior to germination at 20 ºC, followed by pre-incubation at 35 ºC during 2 days prior to germination at 20 ºC, and incubation at different continuous or alternating temperatures; (ii) addition of gibberellic acid in the medium (500 ppm) and germination at 20 ºC; (iii) erosion with sea sand through a small hole made with the hot tip of a fine soldering iron, followed by immersion in liquid nitrogen for 15 min.

Germination was recorded at the 3rd and 11th days from sowing, and the germination percentage was calculated as the percentage of viable seedlings. Our optimum germination conditions were considered as germination percentages higher than 70%, which is the validation criterion commonly used in phytotoxicity tests (OECD 208).

### Phytotoxicity Assays

Phytotoxicity tests were performed on those plant species for which optimum germination conditions were established.

Basically, nominal concentrations of 1, 5, 10, 50, 100, 500 and 1000 mg/L of oxytetracycline and 1, 5, 10, 50, 100 and 500 mg/L of sulfamethazine were independently assayed. Two antibiotic replicates per concentration and two replicates for the control, containing 25 seeds per replicate, were prepared. The experiments were conducted in Petri dishes with filter paper moistened with 6–21 mL of stock solution, in accordance with the determinations set out in the germination conditions (item 2.2). The test solution for each concentration contained 0.005, 0.01, 0.05, 0.1, 0.5, 1 and 5 mg of antibiotic diluted in 5 mL of water. For the standard controls, the same volume of water, instead of test solution, was applied. The seeds were randomly located on the filter paper after chemical or mechanical treatments, when necessary (Item 2.2).

The Petri dishes were maintained in a germination chamber (light cycle 16:8 h), under appropriate growth conditions for the selected tested species (Sect. “Germination conditions”). Their root elongation was determined between days 3 and 11, in accordance with the germination results of each studied species (Sect. “Germination conditions” and Supplementary Table 3). Plants were scanned and roots were measured using the ImageJ software (Schneider et al. 2012). Toxicity assays were validated according to seed emergence in the control group (at least 70%).

Oxytetracycline and sulfamethazine test solutions were analyzed to verify the nominal concentrations used in the phytotoxicity assays. For each sample, 0.5 mL was diluted with 0.5 mL methanol (50:50 v/v). Further dilutions in methanol/water (50:50, v/v) were prepared to obtain a final concentration of 50 µg/L for sulfamethazine and of 100 µg/L for oxytetracycline in the injection vials. Each vial was spiked with 25 µL of a 1 mg/L internal standard mixture, containing isotopically labeled antibiotics (sulfamethazine-d_4_ and tetracycline-d_6_), to obtain a final concentration of 25 ug/L in the injection vials. Instrumental analysis was carried out with an ultra-high-performance liquid chromatography system (UPLC) using an Acquity HSS T3 column (50 mm × 2.1 mm i.d., 1.7 μm particle size), both from Waters Corporation (Mildford, MA, USA), and using an injection volume of 5 µL. The UPLC system was coupled with a hybrid quadrupole-linear ion trap mass spectrometer (UPLC-5500 QTRAP, Applied Biosystems, Foster City, CA, USA) equipped with a Turbo V electrospray ionization source working in positive ionization mode. Two multiple reaction monitoring (MRM) transitions per compound were recorded using the Scheduled MRM™ algorithm (see Gros et al. 2013 for details). All data were acquired and processed using the Analyst 1.6.3 software. Recovery values were calculated in each sample and used to correct the nominal concentrations of the assays if analytical values showed differences > 20% in comparison with nominal concentration.

The results of antibiotic effects were assessed as the percentage of inhibition on root elongation in comparison with the control. The differences in each assay among the doses of each antibiotic were evaluated by one-way analyses of variance (ANOVA). In case of null hypothesis rejection, the 10% and 50% effect concentrations (EC10 and EC50) of root elongation were calculated by the logit method (OECD 2006). In case of null hypothesis acceptance or of a R Square value lower than 0.7 for the concentration–response curve, the non-observed effect concentrations (NOECs) value was determined as the highest tested concentration for which there was no statistically significant difference of effect (p < 0.05) when compared to the control group.

### Sensitivity Analysis

Differences in sensitivity were explored using the EC10 and EC50 datasets. To maximize statistical power, the NOECs were converted into EC10 values by multiplying them by two (Christl et al., 2018).

Statistical differences between crop and wild plant species were assessed using the Mann–Whitney nonparametric test, according to the non-normality of the distribution, previously assessed through the Shapiro–Wilk test. All the statistical tests were performed with a confidence level of 95%.

Alternatively, the predicted no-effect concentration (PNEC) value for crop and wild species was calculated following the Scientific guideline on Plant testing strategy in the risk assessment for veterinary medicinal products (EMA 2017). The PNEC is the concentration of a chemical that establishes the limit below which, in an ecosystem, no adverse effects of exposure are observed. It is based on the percentile 5 estimation of the species sensitivity distribution (SSD) of the ECx dataset for a minimum of eight data, to obtain the reference value that can protect 95% of the species. The percentile or Hazardous Concentration 5 (HC5) was calculated using the ETX 2.0 software (Van Vlaardingen et al. 2004).

## Results

### Optimum Germination Conditions

Overall, 19 out of the 24 studied species showed optimum germination conditions (germination percentage higher than 70%) at 4–7 days after seedling (Supplementary Table 3). Common foxglove was the species that required the longest period (11 days). Timothy grass, sterile oat and scarlet pimpernel required chemical treatment to achieve these optimum germination conditions. The germination results at various temperatures were similar, indicating that assays could be carried out at a standard temperature of 20 °C, except for red clover, which required a temperature of 15 ºC.

The resulting list of plants to be assayed for phytotoxicity (Supplementary Table 3) included 10 crop species, comprised into four families (Asteraceae [n = 2]), Brassicaceae [n = 2], Fabaceae [n = 2]), Poaceae [n = 4]), and nine wild plants of six families (Asteraceae [n = 2], Fabaceae [n = 2], Plantaginaceae [n = 1], Poaceae [n = 2], Polygonaceae [n = 1], and Primulaceae [n = 1]).

### Phytotoxicity Assays

Most of the plant assays were validated according to the germination criteria. Only alfalfa and timothy grass assays with oxytetracycline (one crop species and one wild species) (Table 1), and timothy grass assays with sulfamethazine (wild species) (Table 2), showed germination percentages lower than 70%. The lettuce assay with sulfamethazine showed unforeseen effects that cannot be linked to a dose–response relationship but rather to an artifact within the assay. The lettuce assay with sulfamethazine revealed unexpected effects that cannot be attributed to a dose–response relationship, but rather to an artifact within the assay. In the *Lactuca* bioassay, the control showed a germination rate of 92%, indicating normal behavior. Nevertheless, no linear decrease in germination was observed with increasing concentrations of the antibiotic. The observed effect consistently remained between 22.4% and 25.8%. This contrasts with findings from Pan and Chu (2016), who reported that sulfamethoxazole (SMZ) inhibits root growth in lettuce, with a NOEC of 0.1 mg/L, an EC10 of 1.94 mg/L, and an EC50 of 157 mg/L, and considered lettuce a good biomonitor for veterinary antibiotics in the environment.

**Table 1:** Results of the phytotoxicity test with oxytetracycline: % germination for the control, % effect observed between minimum and maximum concentration assayed and results of the ANOVA test (95% confidence level)

GROUP	CLASS	FAMILY	SPECIES	% GERMINATION	%effect	F-value	p-value
Crop	Monocotyledoneae	Poaceae	*Avena sativa* (oat)	82	28.4–88.5	96.92	< 0.001
			*Hordeum vulgare* (barley)	92	0–54.8	60.96	< 0.001
			*Triticum aestivum* (wheat)	98	13.3–56.5	59.52	< 0.001
			*Zea mays* (maize)	86	6.2–58.9	25.48	< 0.001
	Dicotyledoneae	Asteraceae	*Helianthus annuus* (sunflower)	96	0–71.5	59.67	< 0.001
			*Lactuca sativa* (lettuce)	80	0–44.7	54.04	< 0.001
		Brassicaceae	*Brassica napus* (rapeseed)	86	8.20–83.4	43.43	< 0.001
			*Brassica oleracea* (cabbage)	70	1.6–82.5	27.33	< 0.001
		Fabaceae	*Medicago sativa* (alfalfa)	52	–	–	
			*Vicia sativa* (common vetch)	86	0–61.7	79.38	< 0.001
Wild	Monocotyledoneae	Poaceae	*Avena sterilis* (sterile oat)	92	16.9–78.9	21.34	< 0.001
			*Phleum pratense* (timothy grass)	60	–	–	–
	Dicotyledoneae	Asteraceae	*Bellis perennis* (English daisy)	88	0–71.1	28.14	< 0.001
			*Centaurea cyanus* (cornflower)	96	12.9–58.0	25.14	< 0.001
		Fabaceae	*Lotus corniculatus* (birdsfoot trefoil)	88	11.4–70.8	64.91	< 0.001
			*Trifolium pratense*(red clover)	70	0–42.9	7.97	< 0.001
		Plantaginaceae	*Digitalis purpurea* (purple foxglove)	82	0–100.0	19.94	< 0.001
		Polygonaceae	*Rumex crispus* (curled dock)	98	0–79.4	81.85	< 0.001
		Primulaceae	*Anagallis arvensis* (scarlet pimpernel)	92	0–90.5	64.07	< 0.001

**Table 2:** Results of the phytotoxicity test with sulfamethazine: % germination for the control, % effect observed between minimum and maximum concentration assayed and results of the ANOVA test (95% confidence level)

Group	Class	Family	Species	% Germination	% Effect	F-value	*p*-value
Crop	Monocotyledoneae	Poaceae	*Avena sativa* (oat)	88	0–46.17	21.85	< 0.001
			*Hordeum vulgare* (barley)	92	2.72–28–88	9.93	< 0.001
			*Triticum aestivum* (wheat)	96	0–27.55	16.69	< 0.001
			*Zea mays* (maize)	100	0–8.92	13.21	> 0.05
	Dicotyledoneae	Asteraceae	*Helianthus annuus* (sunflower)	100	1.06–31.95	4.49	> 0.05
			*Lactuca sativa* (lettuce)*	–	–	–	–
		Brassicaceae	*Brassica napus* (rapeseed)	82	0–78.67	24.81	< 0.001
			*Brassica oleracea* (cabbage)	78	20.39–94.31	32.66	< 0.001
		Fabaceae	*Medicago sativa* (alfalfa)	72	0	5.45	> 0.05
			*Vicia sativa* (common vetch)	90	0–12.48	11.60	> 0.05
Wild	Monocotyledoneae	Poaceae	*Avena sterilis* (sterile oat)	80	55.15–79.74	41.38	< 0.001
			*Phleum pratense* (timothy grass)	60	–	–	–
	Dicotyledoneae	Asteraceae	*Bellis perennis* (English daisy)	82	0–55.58	27.47	< 0.001
			*Centaurea cyanus* (cornflower)	88	30.97–72.45	54.26	< 0.001
		Fabaceae	*Lotus corniculatus* (birdsfoot trefoil)	92	25.12–59.36	54.16	< 0.001
			*Trifolium pratense*(red clover)	72	0–3.93	13.33	> 0.05
		Plantaginaceae	*Digitalis purpurea* (purple foxglove)	72	0–86.19	48.75	< 0.001
		Polygonaceae	*Rumex crispus* (curled dock)	98	0–27.47	28.98	< 0.001
		Primulaceae	*Anagallis arvensis* (scarlet pimpernel)	90	21.70–90.99	48.12	< 0.001

^*^No validated. An effect of 20% of toxicity was observed in all assayed concentrations and the control probably due to an artifact

Significant differences between the control and the concentrations assayed were observed in all of the validated plant assays with oxytetracycline (Table 1). Results on sulfamethazine effects showed significant differences for nine of the seventeen validated assays, mostly with wild plant species (Table 2). No significant effects were observed in five species.

The analytical tests (Table 3) showed that oxytetracycline concentrations were within 80–120%, in accordance to the nominal concentration. For sulfamethazine, the measured concentrations did not remain within the 80–120% range. Therefore, the results of the phytotoxicity assays were expressed as nominal concentrations for oxytetracycline and as measured concentrations for sulfamethazine.

**Table 3:** Concentration (mg/L) as mean ± s.d. of oxytetracycline and sulfamethazine used in the germination assays

Nominal concentration (mg L^−1^)	Analytical concentration (mg L^−1^) oxytetracycline	Deviation (%)	Analytical concentration (mg L^−1^) sulfamethazine	Deviation (%)
0	0.0		0.0	
1	0.8 ± 0.03	80	1.4 ± 0.05	135
5	4.0 ± 0.07	80	6.0 ± 0.13	120
10	10.0 ± 0.02	100	12.0 ± 0.83	120
50	49.0 ± 3.40	98	64.5 ± 1.85	129
100	102.0 ± 1.30	102	125.0 ± 2.40	125
500	505.0 ± 16.00	101	495.0 ± 21.50	99
1000	1025.0 ± 1.40	103	–	–

A significant dose–response relationship was observed in all plants exposed to oxytetracycline (OTC), except for sterile oat (Table 4). Toxicity values were within the range of 0.39–26.64 mg/L (n = 9) for EC10_OTC_ and 18.0–846.78 mg/L (n = 9) for EC50_OTC_ in crop species, and 0.18–64.34 mg/L (n = 8) for EC10_OTC_ and 46.02–2611.49 mg/L (n = 8) for EC50_OTC_ in wild species (Table 4; Supplementary Fig. 1). For sulfamethazine (SMZ) (Table 5; Supplementary Fig. 1), a significant dose response relationship was also observed in wild plants (n = 7) with EC10_SMZ_ values in the range of 0.01–69.52 mg/L and EC50 values of 5.01–1672.64 mg/L. Crop species showed dose dependence only for three species (EC10_SMZ_ = 0.89–42.64 mg/L and EC50_SMZ_ = 14.02–847.38 mg/L). Other crop and wild species showed NOEC values within the range of 65–495 mg/L (n = 7).

**Table 4:** Results of the dose–response curve for oxytetracycline: omnibus test (p 0.05) result, R2, EC10 (mg/L) and EC50 (mg/L) (and their respective lower confident limit (LCL) and upper confident limit (UCL)) values. NOEC values (mg/L) are included for those plant species without statistically significant dose–response curve effect. Results are expressed as nominal values

Group	Class	Family	Species	*p*-value	R2	EC10	LCL	UCL	EC50	LCL	UCL	NOEC
Crop	Monocotyledoneae	Poaceae	*Avena sativa* (oat)	5.7E–17	0.87	0.39	0.00	2.03	18.02	4.63	50.61	–
			*Hordeum vulgare* (barley)	2.4E–11	0.81	2.08	0.01	10.41	556.62	147.86	17,900.57	–
			*Triticum aestivum* (wheat)	1.6E–11	0.91	1.33	0.21	3.88	466.32	215.57	1534.06	–
			*Zea mays* (maize)	3.0E–10	0.89	3.04	0.52	8.26	846.78	352.18	3723.91	–
Crop	Dicotyledoneae	Asteraceae	*Helianthus annuus* (sunflower)	4.6E–27	0.88	6.19	0.39	19.86	151.43	57.96	588.23	–
			*Lactuca sativa* (lettuce)	2.0E–16	0.77	26.64	0.08	104.40	782.31	203.58	167,332.61	–
		Brassicaceae	*Brassica napus* (rapeseed)	2.6E–14	0.80	0.50	0.00	3.59	54.57	12.03	388.15	–
			*Brassica oleracea* (cabbage)	8.7E–20	0.93	1.36	0.21	3.78	45.98	22.50	101.57	–
		Fabaceae	*Vicia sativa* (common vetch)	2.3E–20	0.87	9.14	0.68	28.05	289.01	109.59	1646.55	–
Wild	Monocotyledoneae	Poaceae	*Avena sterilis* (sterile oat)	3.0E–05	0.64	–	–	–	–	–	–	1*
	Dicotyledoneae	Asteraceae	*Bellis perennis* (English daisy)	6.5E–26	0.87	17.82	1.37	50.62	331.48	130.90	1807.36	–
			*Centaurea cyanus* (cornflower)	1.2E–05	0.93	0.18	0.00	2.24	1449.49	250.31	861,057.79	–
		Fabaceae	*Lotus corniculatus* (birdsfoot trefoil)	1.0E–16	0.91	2.38	0.23	7.58	178.84	76.65	637.82	–
			*Trifolium pratense* (red clover)	1.1E–09	0.92	64.34	22.63	126.47	2611.49	1013.46	17,667.28	–
		Plantaginaceae	*Digitalis purpurea* (purple foxglove)	1.2E–34	0.98	21.59	12.14	30.25	63.98	48.43	88.69	–
		Polygonaceae	*Rumex crispus* (curled dock)	1.4E–40	0.95	24.12	7.58	46.75	186.79	108.34	348.83	–
		Primulaceae	*Anagallis arvensis* (scarlet pimpernel)	7.0E–35	0.92	5.00	0.67	12.80	46.02	19.93	105.24	–

^*^ Adjustment of the dose–response curve lower than OECD 208 criteria (R2 ≥ 0.7)

**Table 5:** Results of the dose–response curve for sulfamethazine: omnibus test (p 0.05) result, R2, EC10 (mg/L) and EC50 (mg/L) (and their respective lower confident limit (LCL) and upper confident limit (UCL)) values: % germination for the control, % effect observed between minimum and maximum concentration assayed and results of the ANOVA test (p 0.05)

Group	Class	Family	Species	*p*-value	R^2^	EC10	LCL	UCL	EC50	LCL	UCL	NOEC
Crop	Monocotyledoneae	Poaceae	*Avena sativa*	3.4E–17	0.86	42.64	4.48	116.92	847.38	296.20	11,520.77	–
			*Hordeum vulgare*	4.4E–05	0.36	–	–	–	–	–	–	125*
			*Triticum aestivum*	7.5E–07	0.65	–	–	–	–	–	–	65*
			*Zea mays*	> 0.05	–	–	–	–	–	–	–	495**
	Dicotyledoneae	Asteraceae	*Helianthus annuus*	> 0.05	–	–	–	–	–	–	–	495**
		Brassicaceae	*Brassica napus*	2.1E–17	0.94	6.41	1.32	14.61	111.70	56.91	299.14	–
			*Brassica oleracea*	6.7E–21	0.95	0.89	0.12	2.43	14.02	6.53	27.55	–
		Fabaceae	*Medicago sativa*	> 0.05	–	–	–	–	–	–	–	495**
			*Vicia sativa*	> 0.05	–	–	–	–	–	–	–	495**
Wild	Monocotyledoneae	Poaceae	*Avena sterilis*	8.2E–03	0.94	0.01	1.9E–26	0.54	5.01	1.1E–06	21.66	–
	Dicotyledoneae	Asteraceae	*Bellis perennis*	2.0E–16	0.77	27.17	9.7E–05	100.06	375.43	101.9	2.0E + 08	–
			*Centaurea cyanus*	2.6E–07	0.73	0.13	1.6E–16	2.17	59.99	8.04	25,653.58	–
		Fabaceae	*Lotus corniculatus*	6.7E–06	0.88	0.07	4.6E–04	0.52	79.48	33.67	292.41	–
			*Trifolium pratense*	> 0.05	–	–	–	–	–	–	–	495**
		Plantaginaceae	*Digitalis purpurea*	1.1E–19	0.94	4.11	0.78	8.99	29.82	15.19	63.41	–
		Polygonaceae	*Rumex crispus*	1.2E–15	0.73	69.52	3.5E + 10	790.44	1672.64	294.51	4.1E–44	–
		Primulaceae	*Anagallis arvensis*	1.4E–17	0.76	2.35	0.18	22.74	26.12	#N/A	#N/A	–

^*^No effect compared to the control according to ANOVA test (see Table 6)

^**^Adjustment of the dose-curve response lower than OECD 208 criteria (R^2^ ≥ 0.7)

NOEC values (mg/L) are included for those plant species without statistically significant dose–response curve effect. Results are expressed as analytical values

### Sensitivity Analysis

**Fig. 1:**
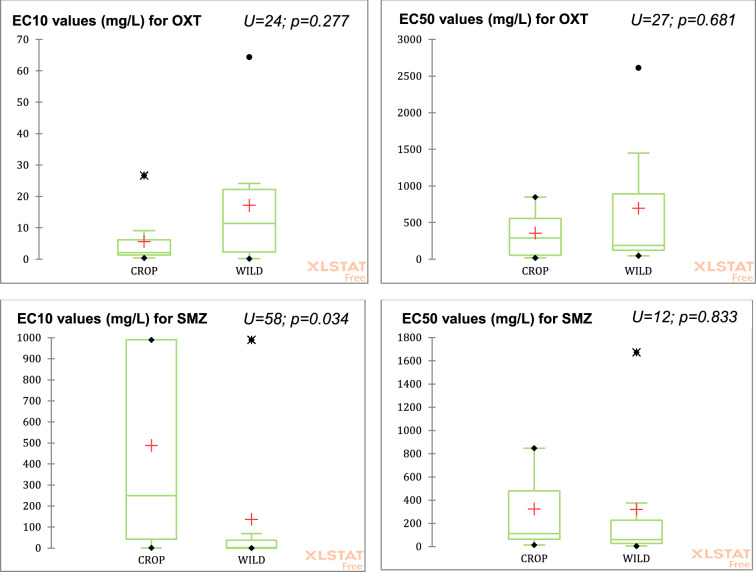
Box-plot and Mann–Whitney test results of the toxicity ECx values (mg/L) for oxytetracycline (OXT) and sulfamethazine (SMZ)

The results of the sensitivity analysis for oxytetracycline (Fig. 1) did not show statistical differences between the averages of crop and wild species regarding neither EC10_OTC_ (U = 24; p = 0.227) nor EC50_OTC_ (U = 27; p = 0.681). By contrast, the sulfamethazine results for EC10_SMZ_ (U-value = 57.5; p = 0.034) indicated that wild plants are significantly more susceptible than crops (Fig. 1). No statistical differences for EC50SMZ were observed (U = 12; p = 0.833).

**Fig. 2:**
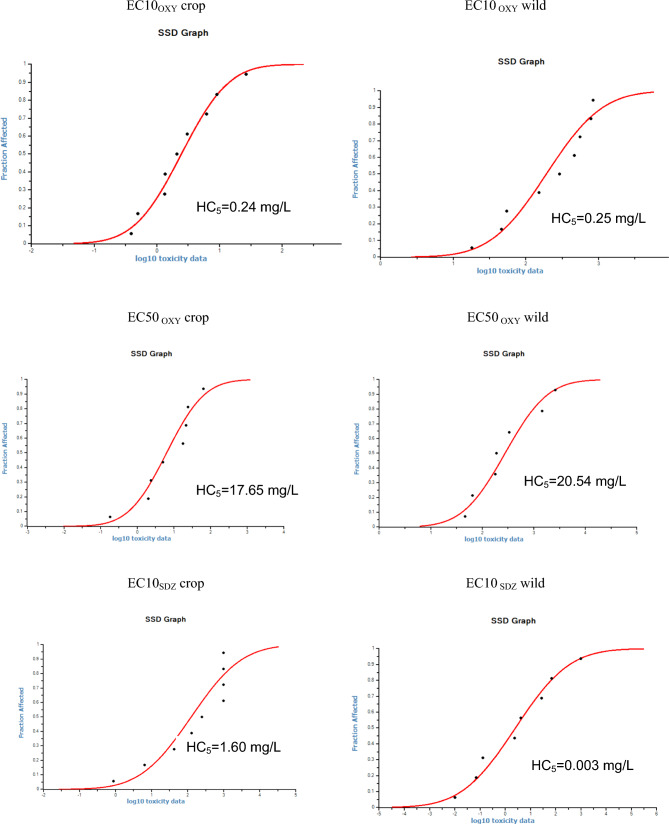
Dose–response curve and HC5 values of the log toxicity ECx data (mg/L) for oxytetracycline (OXT) and sulfamethazine (SMZ)

The calculation of the PNEC values (hazardous concentration HC5) also showed a high sensitivity of wild plants to sulfamethazine (0.003 wild *vs.* 1.60 crop for EC10_SMZ_) (Fig. 2). For oxytetracycline, HC5 values for crop and wild plants were closely located (Fig. 2) (0.24 *vs* 0.25 for EC10_OTC_; 17.65 *vs.* 20.54 for EC50_OTC_).

## Discussion

The detrimental effects of antibiotics in crop plants have been intensively studied; however, their impacts on wild species have received much less attention (Minden et al. 2017; Carballo et al. 2022). Herein, the phytotoxicity tests based on root elongation demonstrated that antibiotics could negatively affect crop and wild plants, but the effects varied depending on plant species and the antibiotics used. Previous antibiotic studies indicated that root elongation was the most sensitive endpoint (Hillis et al. 2011; Liu et al. 2009; Minden et al. 2017). They also suggested that seed coating serves as a protective barrier that isolates the embryo from its surroundings (Radchuk, and Borisjuk 2014). Upon germination, the roots of the emerging seedling absorb contaminants, which potentially affect the subsequent growth of the developing plant, including root elongation (Sanz et al. 2022).

Root elongation of most of the wild species was inhibited in the presence of oxytetracycline and sulfamethazine, whereas crops were mainly affected by oxytetracycline. Among the tested antibiotics, there were no statistical differences in sensitivity to oxytetracycline between crop (EC10_OTC_ 0.39–26.64 mg/L) and wild plant species (EC10_OTC_ 0.18–64.35 mg/L), whereas wild plants were more susceptible to sulfamethazine (EC10_SMZ_ 0.01–990 mg/L) than crop species (EC10_SMZ_ 0.89–990 mg/L).

Our results are consistent with those of previous studies in crop species (Pino et al. 2016; Liu et al. 2009; Timmerer et al. 2020). Liu et al (2009) found that crops (i.e., sweet oat *Cichorium endivia*, rice *Oryza sativa* and cucumber *Cucumis sativus*) were susceptible to sulfonamides (sulfamethoxazole and sulfamethazine) and tetracyclines (chlortetracycline and tetracycline), reporting similar EC10 range values for root elongation than those observed in our experiments: EC10 of 0.2–16 mg/L for tetracyclines and EC10 of 0.1- > 300 mg/L for sulfonamides. Comparable results for EC50 were reported by Pino et al. (2016) and Timmerer et al. (2020), who determined root growth effects in crop species (lettuce and cabbage), reporting EC50 values of 109 mg/L for tetracycline and 9.8–109 mg/L for sulfonamides (sulfadiazine and sulfamethoxazole), respectively. These values were within the data range obtained in the current experiment, with EC50_OTC_ of 18–846.78 mg/L and EC50_SMZ_ 14.02–847.38. Conversely, Hillis et al. (2011) found higher susceptibility to tetracyclines (chlortetracycline, oxytetracycline and tetracycline) and sulfonamides (sulfamethoxazole and sulfamethazine) in crop species (carrot *Daucus carota*, lettuce and alfalfa). In comparison with data previously described, EC10_root elongation_ values from Hillis et al. (2011) were much lower (0.0031–6.73 mg/L for tetracyclines and 0.011- > 10 mg/L for sulfonamides). Specifically, for oxytetracycline, they reported similar toxicity for crop species than those observed in our experiments (EC10_Hillis_ of 0.5–6.7 mg/L *vs* EC10_experiment_ 0.2–16 mg/L). In the case of sulfamethazine, they found lower toxicity (EC10_Hillis_ of 0.1–5.3 mg/L *vs* EC10_experiment_ 0.9–990 mg/L); for instance, in alfalfa, they reported an EC10_OTC_ of 5.3 mg/L, while we did not observe any effect. These differences could be attributed to methodological variations, emphasizing the need to perform sensitivity comparisons among species within the same trials to avoid uncertainties associated with test conditions or methodological differences.

Studies comparing the effects of antibiotics on crop and wild species are very scarce. Minden et al. (2017) studied the effect of sulfadiazine and tetracycline over two crop species (rapeseed *Brassica napus* and wheat *Triticum aestivum*) and two non-crop species (Shepherd's purse *Capsella bursa-pastoris* and common windgrass *Apera spicaventi*), commonly found in field margins. The authors conducted a greenhouse experiment at antibiotic concentrations of 1, 5 and 10 mg/L, and effects on plants were determined at 8 weeks. Plants responded more markedly to sulfadiazine than to tetracycline. Higher toxicity was observed in the two herb species—one wild species (Shepherd's purse) and one crop species (rapeseed); while differences regarding sensitivity in crop vs. wild species were not reported (Minden et al. 2017). Because this study was designed as a long-term experiment (8 weeks), it was not possible to directly compare our results with those found by Minden et al. (2017).

Our study included toxicity data for two antibiotics, on nine crops and eight wild herbaceous species that have been suggested as the most appropriate sentinel plants in assessments of environmental risk for antibiotics in Mediterranean Europe (Garcia et al., 2022). According to Garcia et al. (2022), the selected crop species most commonly belong to the monocot species, which dominate the cropland scenario in the EU (e.g., barley, wheat and corn). The same applies to the dicot species selected in the study, as they are commonly cultivated for oilseed (sunflower and rapeseed) and fodder (vetch and alfalfa) production (Garcia et al., 2022). The selected wild species have been described as the most abundant in pasture lands (e.g., red clover and birdsfoot trefoil), are extremely abundant along most cereal field margins (e.g., sterile oat) or represent the high plant species biodiversity in the Mediterranean Basin—one of Europe’s hotspots in terms of ecosystem services (Schröter et al. 2005).

Herein, crop and wild species showed similar sensitivity against oxytetracycline, while wild plants are significantly more sensitive than crops to sulfamethazine. Minden et al. (2017) conducted a second experiment to evaluate the effects of beta-lactams (penicillin), sulfonamides (sulfadiazine) and tetracyclines (tetracycline) (all at 1 mg, 5 mg and 10 mg antibiotic/L) on two crop species and two non-crop species. Plants showed clear responses (even at low concentrations) to the treatments at later growth stages of germination (e.g., delayed germination and post-germinative development), with more pronounced effects for penicillin and sulfadiazine than for tetracycline. They concluded that (i) plants’ responses depended on the plant species and antibiotic, (ii) the yield of farmland fertilized with manure containing antibiotics may be compromised due to its negative consequences to cropland species, and (ii) that antibiotics can alter the composition of plant species in natural field margins, with unknown consequences for higher trophic levels, like pollinating and herbivorous insects (Ebeling, et al. 2018).

This study also estimated the PNEC value of two antibiotics on early germination to identify differences in wild versus crop plants. In order to do so, we have estimated the HC5 value of the ECx dataset. Our results indicated similar sensitivity against oxytetracycline, while results with sulfamethazine indicated that the studied wild plants are one order of magnitude more sensitive than crops. Additionally, the sulfamethazine response range among wild species was four orders of magnitude higher than the interspecies differences usually reported for both inorganic and organic compounds, which are often limited to one or two orders of magnitude (Wang 1992). Thus, our findings indicate a need to test antibiotics in a setting including different wild plant species than those previously studied. Additionally, they also allow the comparison of antibiotic toxicity between crop and wild species, avoiding differences in sensitivity that could most likely be attributed to variations in the experimental setup.

Due to increasing water scarcity exacerbated by climate change, the use of wastewater for crop irrigation is on the rise. In Spain, for example, approximately 57,142 hectares -about 1% of irrigated land—are currently irrigated with treated wastewater (Ministerio de la Agricultura, Pesca y Alimentación del Gobierno de España (MAPA): https://www.mapa.gob.es/es/desarrollo-rural/temas/gestion-sostenible-regadios/regadio-espanya/default_1.1.1.aspx. Assessed on September 18th, 2024).

Rodriguez-Mozaz et al. (2020) reported mean concentrations of sulfamides (0.142 mg/L) and tetracyclines (0.094 mg/L) from 13 wastewater treatment plants (WWTPs) sampled across seven EU countries. The environmental risk posed by these antibiotics can be evaluated using the risk quotient (RQ), which is the ratio of the predicted environmental concentration (PEC) to the predicted no-effect concentration (PNEC). We can consider the concentrations reported by Rodriguez-Mozaz et al. (2020) as PEC values, while our estimated HC5 values derived from EC10 results can serve as PNEC values.

Using this approach, we calculated an RQ_SMZ_ value of 0.1 for crop species [141.9/(1.6*1000)] and 0.4 [141.9/(3*1000)] for wild species. In regards to oxytetracycline, the RQ_OTC_ value is 0.4 [94/(0.24*1000)] for crop species and 0.4 [94/(0.25*1000)] for wild species. Based on this analysis, sulfamethazine could occasionally pose a risk to wild species. In fact, higher concentrations of tetracyclines and specifically oxytetracycline (up to 1.82 mg/L) have been reported by other authors in European wastewaters and rivers (Antos et al. 2024).

Due to their inherent characteristics, PNEC levels are designed to warrant caution, defining the concentration at which the chemical or compound under consideration would not induce a toxic effect. According to our results, we recommend that the PNEC values of pharmaceuticals should be based on phytotoxicity data from crop and wild plant species in order to cover the two relevant protection goals: productivity and biodiversity.

Nevertheless, it is necessary to perform additional toxicity tests to get further insights into the soil–plant systems, which are closer to natural systems (Timmerer et al. 2020). In soil, antibiotics are subjected to a series of physical, chemical, and biological processes, mainly solubility, adsorption to soil organic matter or clay minerals, and biodegradation (Cycoń et al., 2019). These processes depend on the physicochemical properties of the antibiotics, the characteristics of the soil, and weather conditions (Conde-Cid et al., 2020). Sulfonamides present high solubility, low adsorption, and low reactivity with soil particles, making them highly mobile in soils (Conde-Cid et al. 2019). Tetracyclines have larger soil sorption coefficients and are relatively immobile in the soil, mainly driven by the organic matter, multivalent cation, clay contents, and the pH of soils (Conde-Cid et al., 2020).

In summary, herein we observed that the phytotoxic effect of oxytetracycline over the elongation of crop and wild plant species was similar, while sulfamethazine effects were more significant in wild plants when compared to crops. Although direct phytotoxicity of antibiotics over plant growth can be estimated by using the early germination test, further plant growth experiments evaluating antibiotic exposure in soil are required to study their effect in a scenario where adsorption, as well as degradation, can occur.

## Supplementary Information

Below is the link to the electronic supplementary material.

Supplementary file1 (DOCX 1118 KB)

## Data Availability

All data generated or analyzed during this study are included in this article and its supplementary files.
