# Supplementary material for: Comparison of Oxytetracycline and Sulfamethazine Effects Over Root Elongation in Selected Wild and Crop Plants Commonly Present in the Mediterranean Cropland and Pasture Scenarios
Source: Arch Environ Contam Toxicol. 2024 Dec 7;88(1):97–109. doi: 10.1007/s00244-024-01104-7 (PMC11782457; doi:10.1007/s00244-024-01104-7)

**Supplementary** **Table 1**. Physiochemical properties of the selected veterinary antibiotics and their respective chemical structures. K_OW_: distribution coefficient octanol-water. pKa: negative log of the acid dissociation constant.

| **Antibiotic** | **Chemical group** | **Chemical formula** | **Molecular weight (g mol_-1_)** | **Water solubility (mg mL_-1_)** | **Log K_OW_ ^a^** | **pKa ^b^** |
| --- | --- | --- | --- | --- | --- | --- |
| oxytetracycline hydrochloride | Tetracyclines | [C](https://pubchem.ncbi.nlm.nih.gov#query=C22H24N2O9)_[22](https://pubchem.ncbi.nlm.nih.gov#query=C22H24N2O9)_[H](https://pubchem.ncbi.nlm.nih.gov#query=C22H24N2O9)_[24](https://pubchem.ncbi.nlm.nih.gov#query=C22H24N2O9)_[N](https://pubchem.ncbi.nlm.nih.gov#query=C22H24N2O9)_[2](https://pubchem.ncbi.nlm.nih.gov#query=C22H24N2O9)_[O](https://pubchem.ncbi.nlm.nih.gov#query=C22H24N2O9)_[9](https://pubchem.ncbi.nlm.nih.gov#query=C22H24N2O9)_ | 460.4 | 1.4 | -0.90 | 3.3, 7.3 |
| sulfamethazine | Sulfonamides | C_12_H_14_N_4_O_2_S | 278.33 | 0.23 | 0.89 | 2.0, 7.49 |

^a^ Conde-Cid et al., 2020

^b^ Drugbank.com

**Supplementary Table 2.** Seed origin of the assayed plant species.

| **GROUP** | **CLASS** | **FAMILY** | **SPECIES** | **SEED ORIGIN** |
| --- | --- | --- | --- | --- |
| Crop | Monocotyledoneae | Poaceae | *Avena sativa* var*. Chapela* (oat) | Harvested at Finca La Canaleja (Madrid, Spain), INIA/CSIC, 2020. |
|  |  |  | *Hordeum vulgare* var*. Vinagrosa* (barley) | Harvested at Finca La Canaleja (Madrid, Spain), INIA/CSIC, 2016. |
|  |  |  | *Triticum aestivum* var*. Aragón* (wheat) | Plant genetic resources base collection, INIA/CSIC, 2015, BGE 00856. |
|  |  |  | *Zea mays* var*. Serraeus* (maize) | Donation from the Biological Mission of Galicia (Spain), CSIC. |
|  | Dicotyledoneae | Asteraceae | *Helianthus annuus* var. *Shakira* (sunflower) | Donation from a farmer's harvest in 2021, Sisamón (Zaragoza, Spain). |
|  |  |  | *Lactuca sativa* var. *Maravilla 4 seasons* (lettuce) | Intersemillas, batch number XISTT3659S00. |
|  |  | Brasicaceae | *Brassica napus* (rapeseed) | Donation from Agrosa semillas selectas, S.A. |
|  |  |  | *Brassica oleracea* var*. Asa de Cántaro* (cabbage) | Rocalba, S.A. batch number 109972. |
|  |  | Fabaceae | *Medicago sativa* (alfafa) | Semillas Batlle, SA, batch number RO3350. |
|  |  |  | *Vicia sativa* var*. Aitana* (common vetch) | Industrias Agrarias Castellanas, SA, batch number ES-07-13-0048/9316. |
| Wild | Monocotyledoneae | Poaceae | *Avena sterilis* (sterile oat) | Harvested at Finca La Canaleja (Madrid, Spain), INIA/CSIC, 2012. |
|  |  |  | *Echinochloa crus-galli* | Semillas Silvestres, S.L. Cultivated, batch number 10360. |
|  |  |  | *Phleum pratense* (timothy grass) | Semillas Cantueso, cultivation origin in Italy, batch number 21117. |
|  | Dicotyledoneae | Asteraceae | *Bellis perennis* (English daisy) | Semillas Silvestres, S.L. Cultivated. Batch number 22680. |
|  |  |  | *Centaurea cyanus* (cornflower) | Semillas Silvestres, S.L. Cultivated. Batch number 22440. |
|  |  | Brasicaceae | *Cardamine pratensis* (bittercress) | Saflax, batch number 15233. |
|  |  | Fabaceae | *Lotus corniculatus* (birdsfoot trefoil) | Semillas Silvestres, S.L. Cultivated. Batch number 22630. |
|  |  |  | *Trifolium pratense* (red clover) | Semillas Cantueso, batch number 18077. |
|  |  | Hypericaceae | *Hypericum perforatum* (St. John’s worth) | Semillas Cantueso, batch number 20265. |
|  |  | Papaveraceae | *Papaver rhoeas* (common poppy) | Semillas Cantueso, batch number 20176. |
|  |  | Plantaginaceae | *Digitalis purpurea* (purple foxglove) | Semillas Cantueso, batch number 11260. |
|  |  | Polygonaceae | *Rumex crispus* (curled dock) | Semillas Silvestres, S.L., batch number 21790. |
|  |  | Primulaceae | *Anagallis arvensis* (scarlet pimpernel) | Semillas Cantueso, batch number 20219. |
|  |  | Rubiaceae | *Gallium aparine* (cleaver) | Semillas Silvestres, S.L. Cultivo, batch number 20590. |

**Supplementary Table 3.** Days required for germination at the growth conditions implemented in this study.

| **GROUP** | **CLASS** | **FAMILY** | **SPECIES** | **GERMINATION (%)** | **DAYS FOR GERMINATION** | **Tª (ºC)** | **ADDITIONAL TREATMENT** |
| --- | --- | --- | --- | --- | --- | --- | --- |
| Crop | Monocotyledoneae | Poaceae | *Avena sativa* var*. Chapela* (oat) | 90 | 7 | 20 |  |
|  |  |  | *Hordeum vulgare* var*. Vinagrosa* (barley) | 100 | 4 | 20 |  |
|  |  |  | *Triticum aestivum* var*. Aragón* (wheat) | 96 | 4 | 20 |  |
|  |  |  | *Zea mays* var*. Serraeus* (maize) | 94 | 6 | 20/30 |  |
|  | Dicotyledoneae | Asteraceae | *Helianthus annuus* var. *Shakira* (sunflower) | 96 | 5 | 20 |  |
|  |  |  | *Lactuca sativa* var. *Maravilla 4 seasons* (lettuce) | 88 | 3 | 20 |  |
|  |  | Brasicaceae | *Brassica napus* (rapeseed) | 86 | 4 | 20 |  |
|  |  |  | *Brassica oleracea* var*. Asa de Cántaro* (cabbage) | 84 | 7 | 20 |  |
|  |  | Fabaceae | *Medicago sativa* (alfafa) | 90 | 4 | 20 |  |
|  |  |  | *Vicia sativa* var*. Aitana* (common vetch) | 98 | 4 | 20 |  |
| Wild | Monocotyledoneae | Poaceae | *Avena sterilis* (sterile oat) | 90 | 7 | 20 | Giberelic acid |
|  |  |  | *Phleum pratense* (timothy grass) | 84 | 7 | 20 | Giberelic acid |
|  | Dicotyledoneae | Asteraceae | *Bellis perennis* (English daisy) | 94 | 6 | 20 |  |
|  |  |  | *Centaurea cyanus* (cornflower) | 96 | 4 | 20 |  |
|  |  | Fabaceae | *Lotus corniculatus* (birdsfoot trefoil) | 89 | 6 | 20 |  |
|  |  |  | *Trifolium pratense* (red clover) | 87 | 6 | 15 |  |
|  |  | Plantaginaceae | *Digitalis purpurea* (purple foxglove) | 96 | 11 | 20 |  |
|  |  | Polygonaceae | *Rumex crispus* (curled dock) | 100 | 7 | 20 |  |
|  |  | Primulaceae | *Anagallis arvensis* (scarlet pimpernel) | 93 | 9 | 20 | Giberelic acid |

**Supplementary Figure 1.** Dose-response curves and ANOVA graphics for oxytetracycline (left) and sulfamethazine (right).


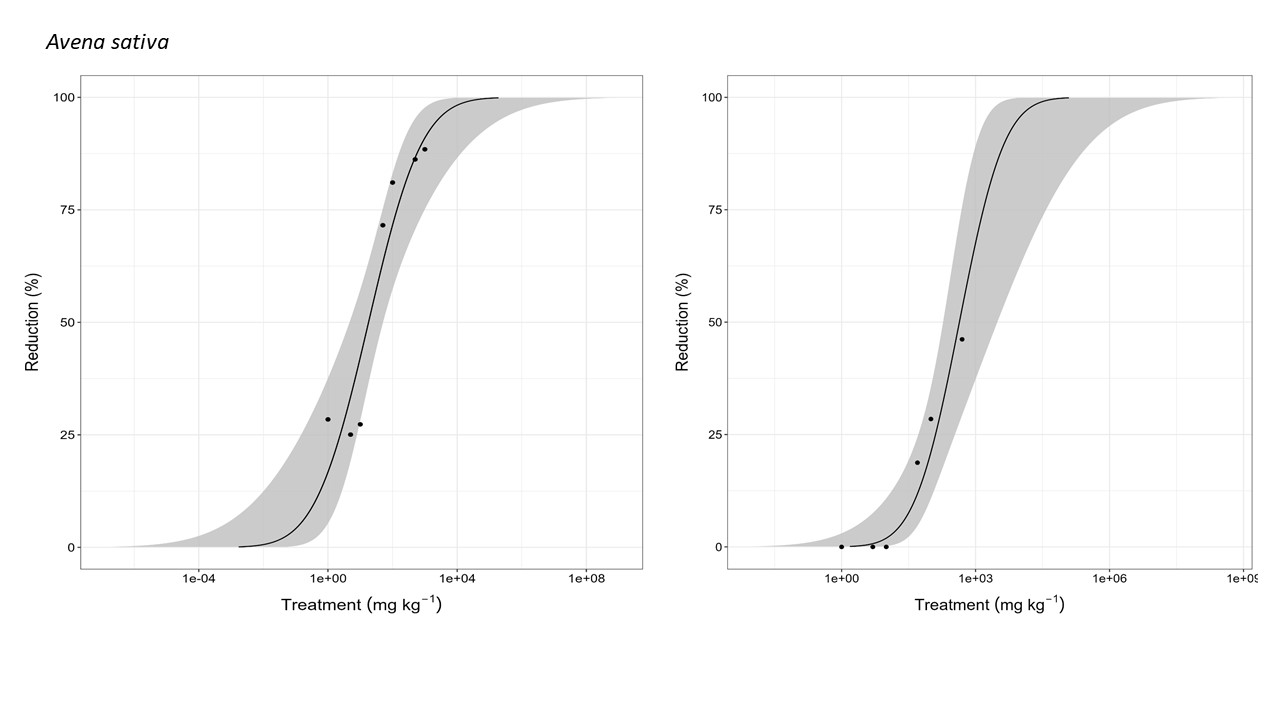


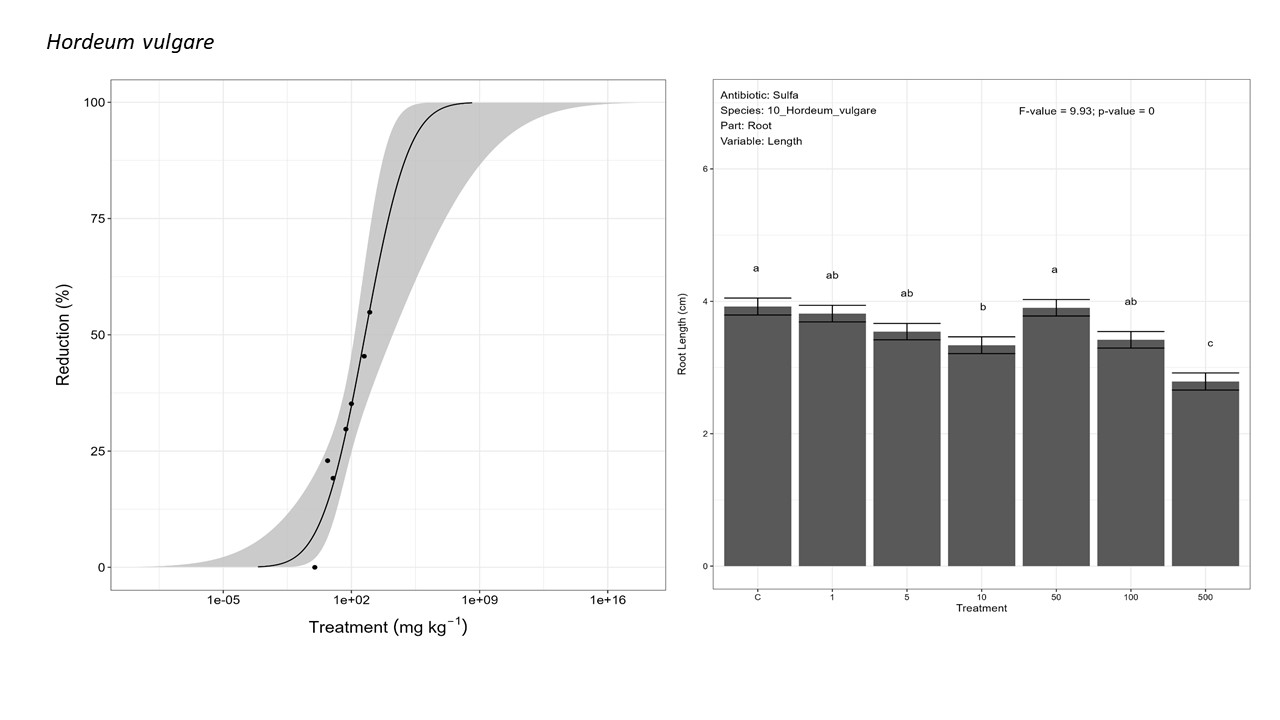

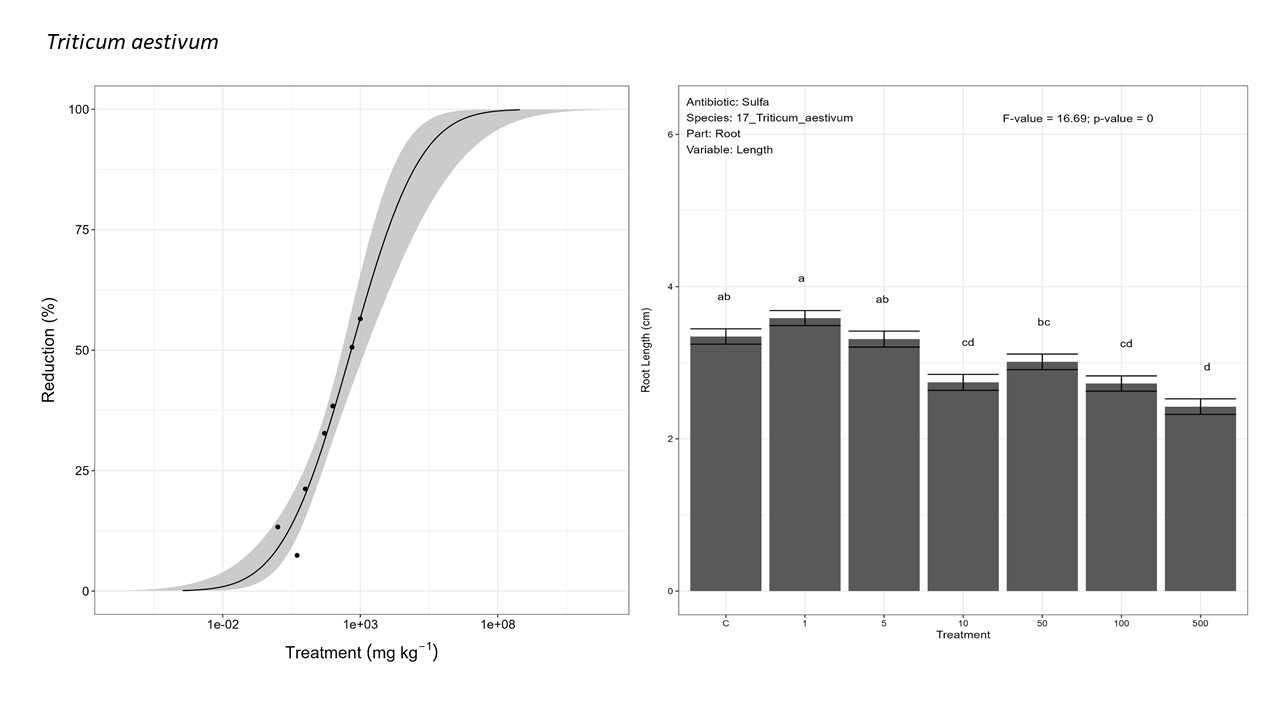

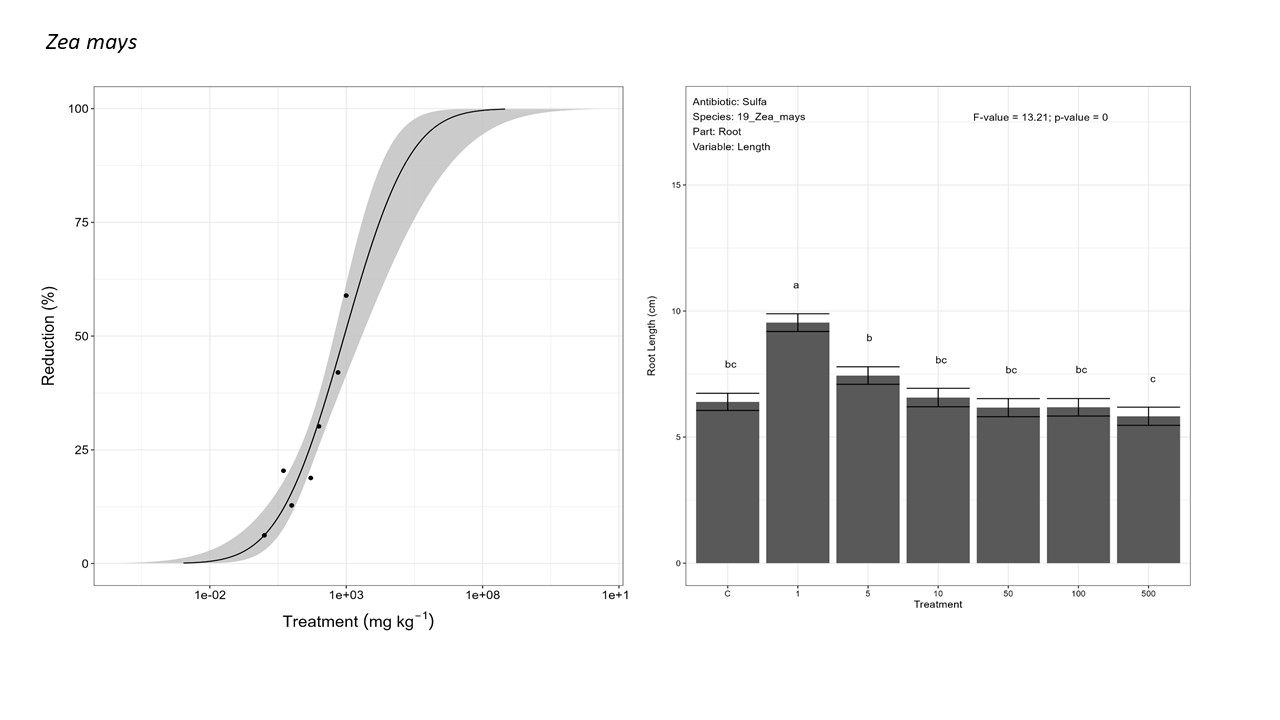

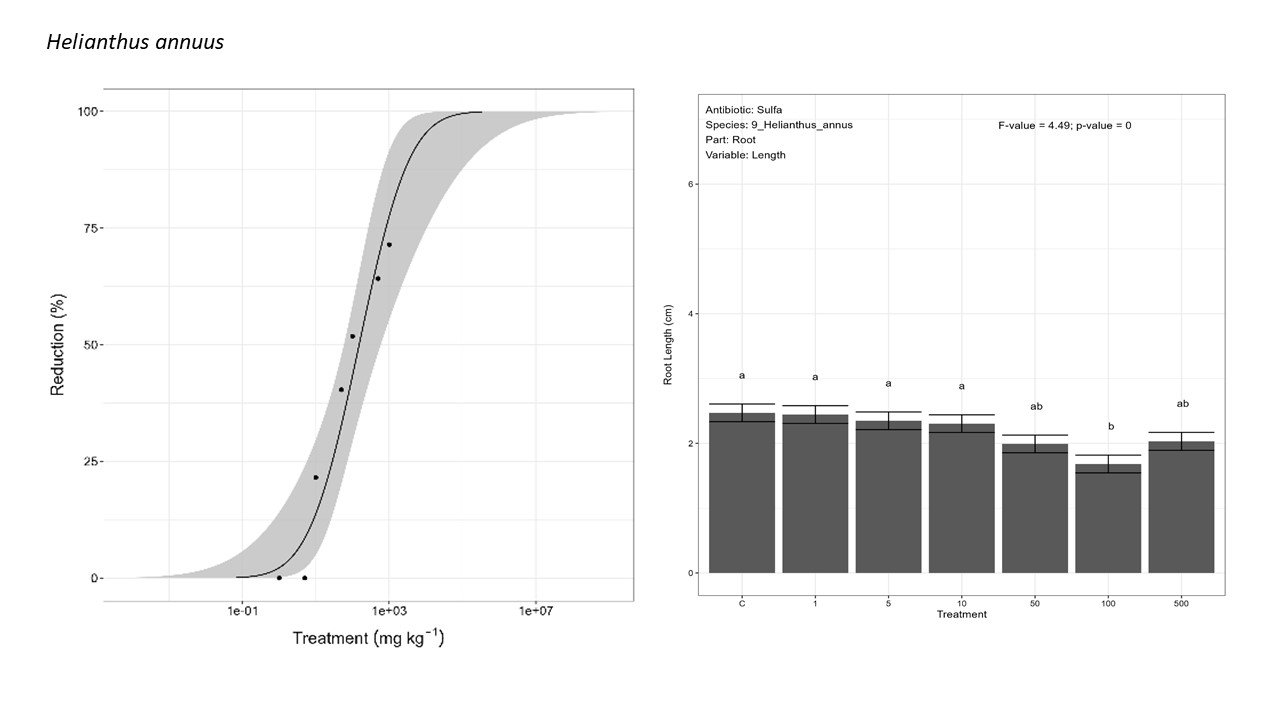

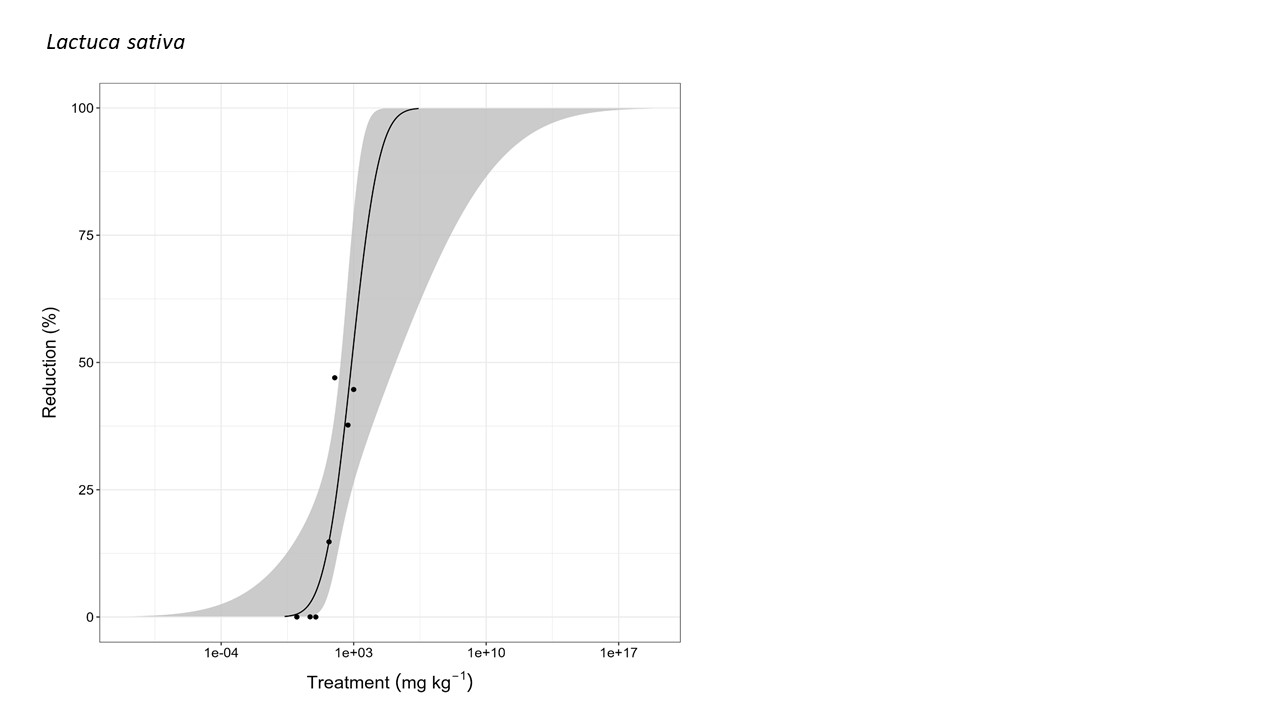

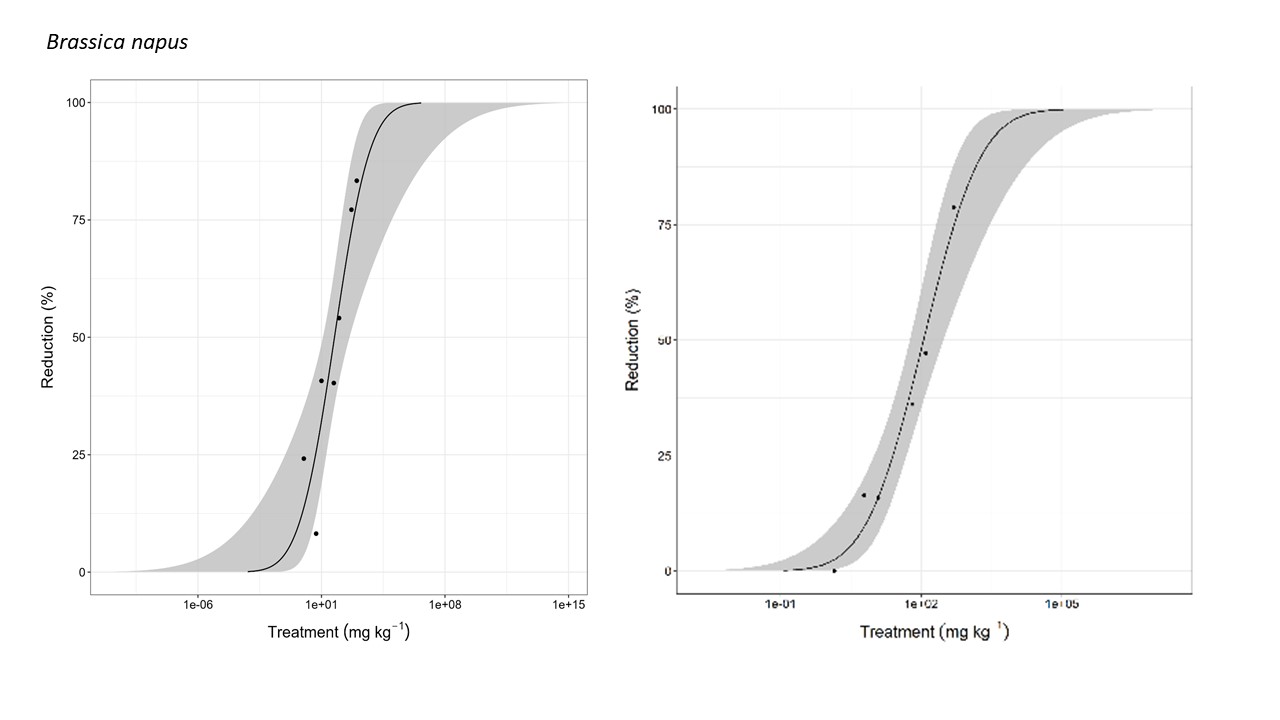

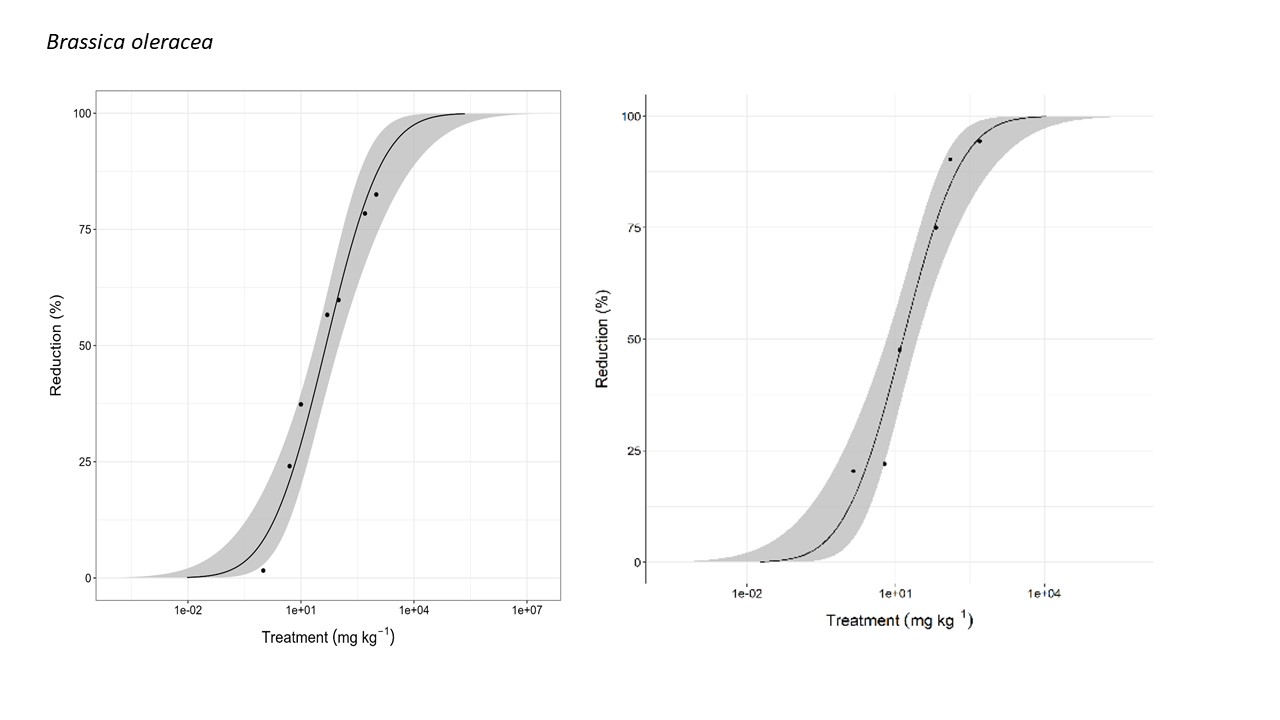

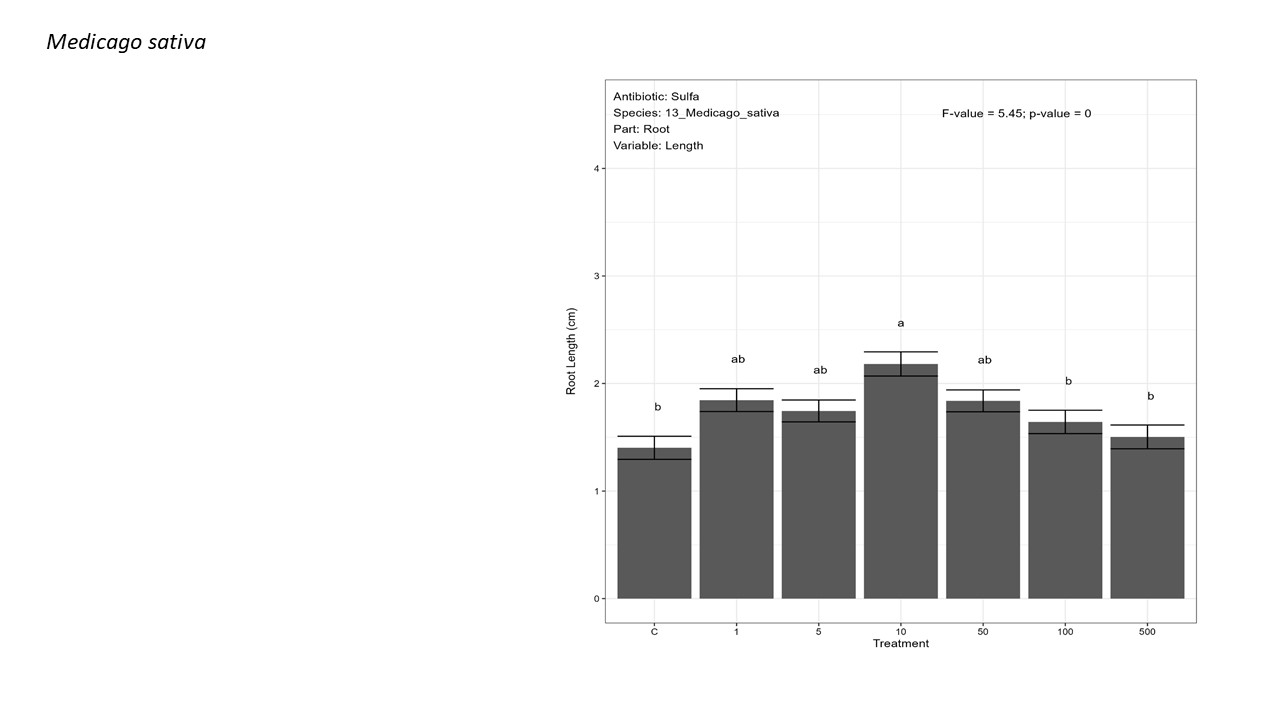


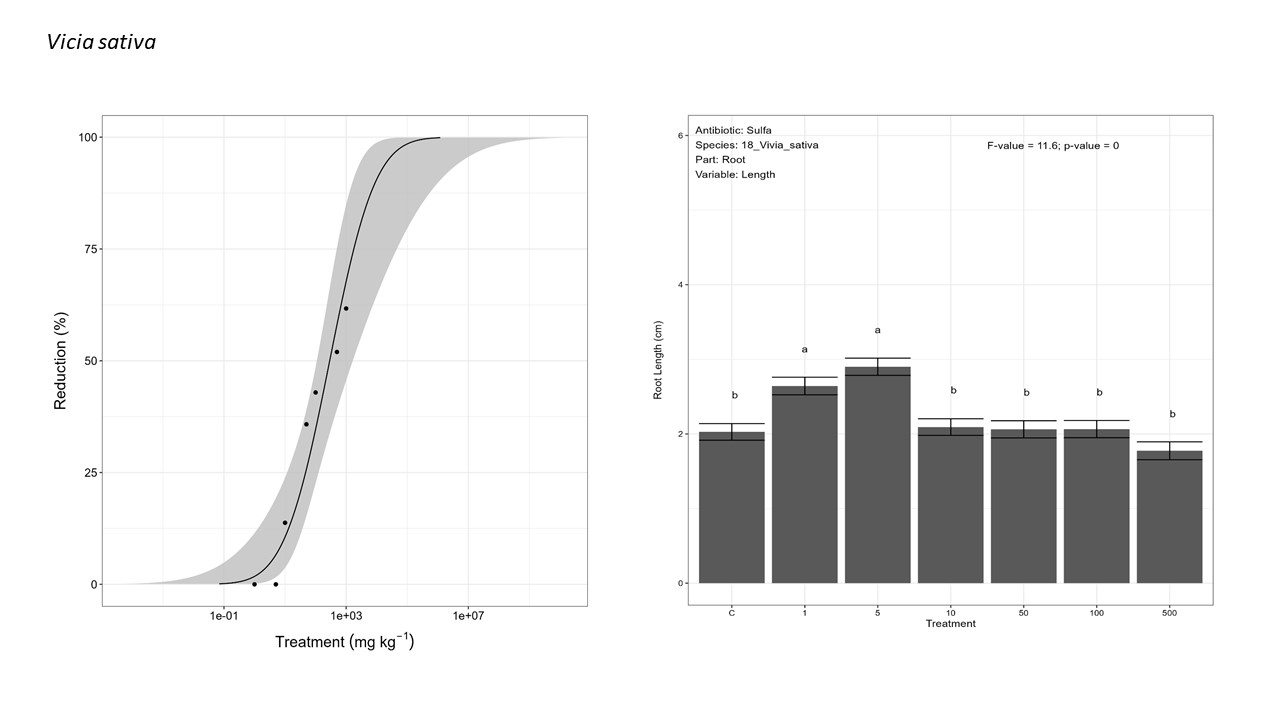


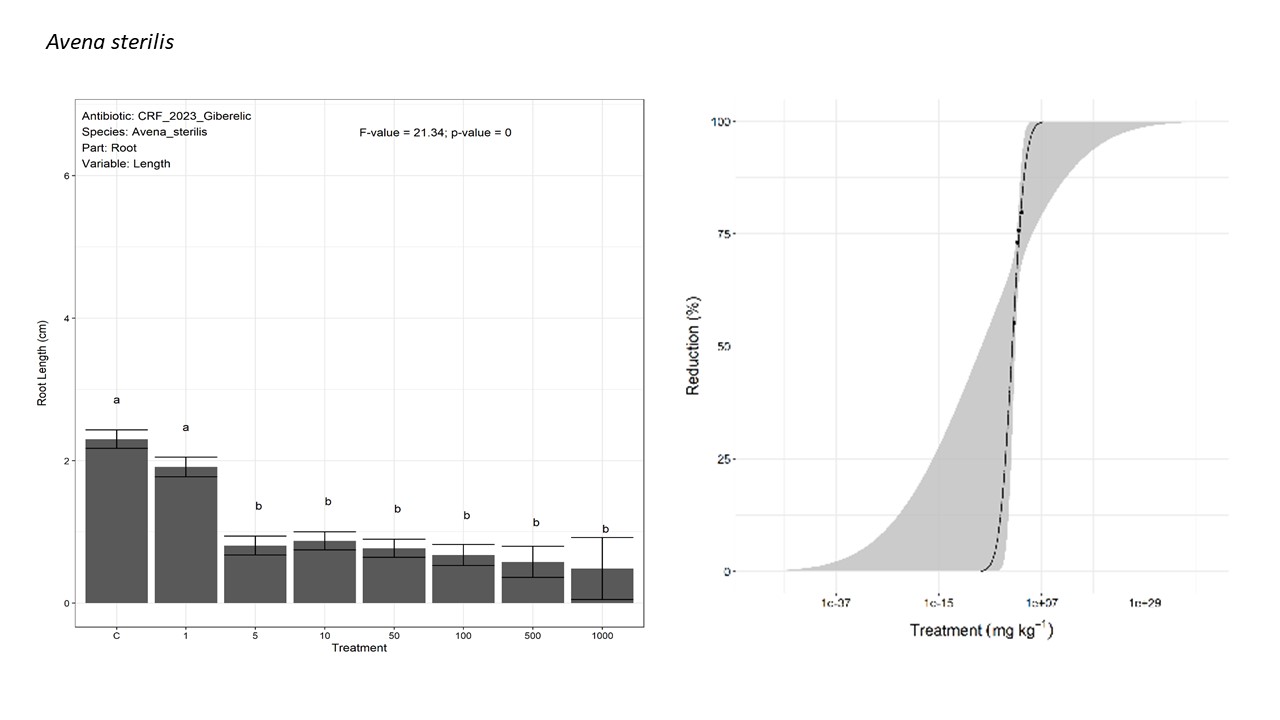

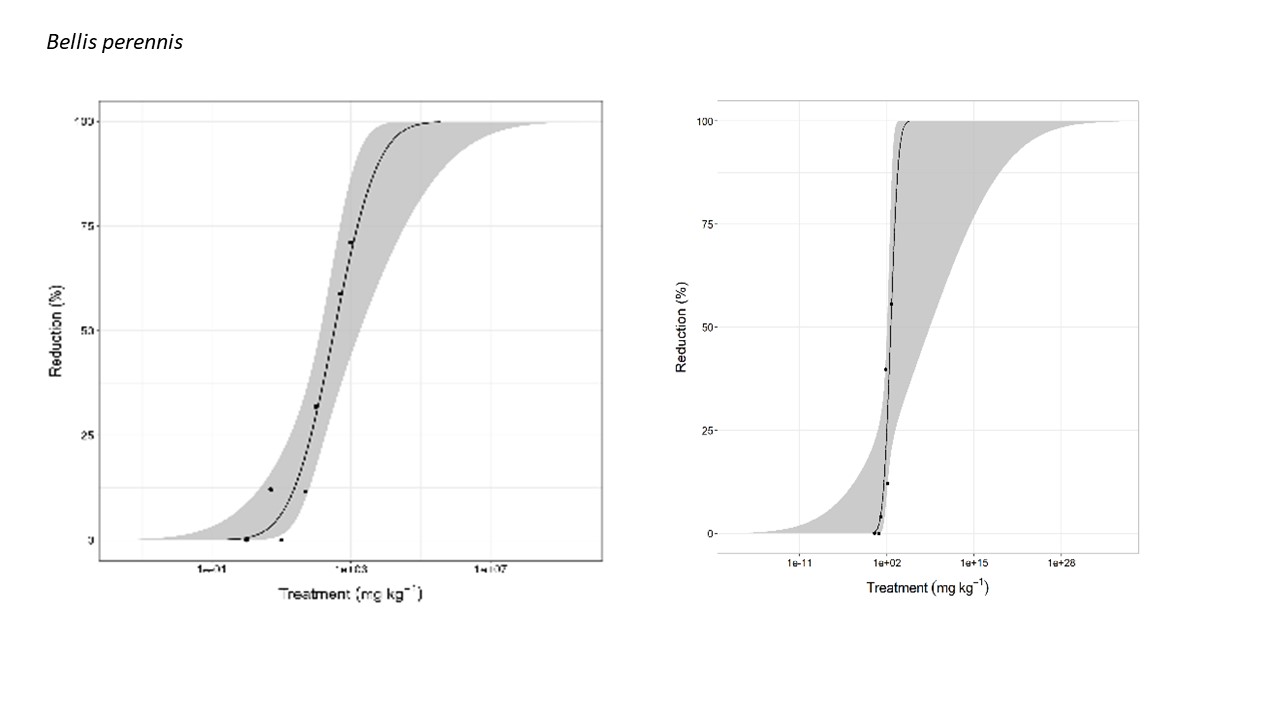

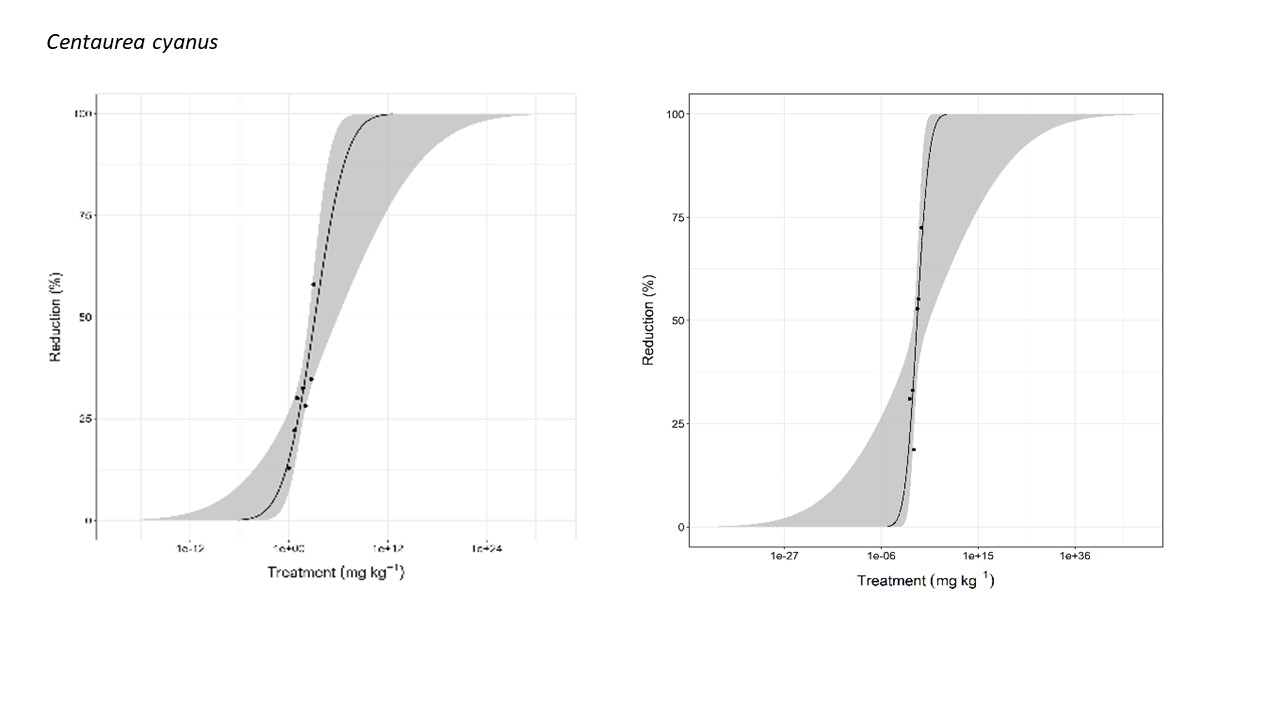

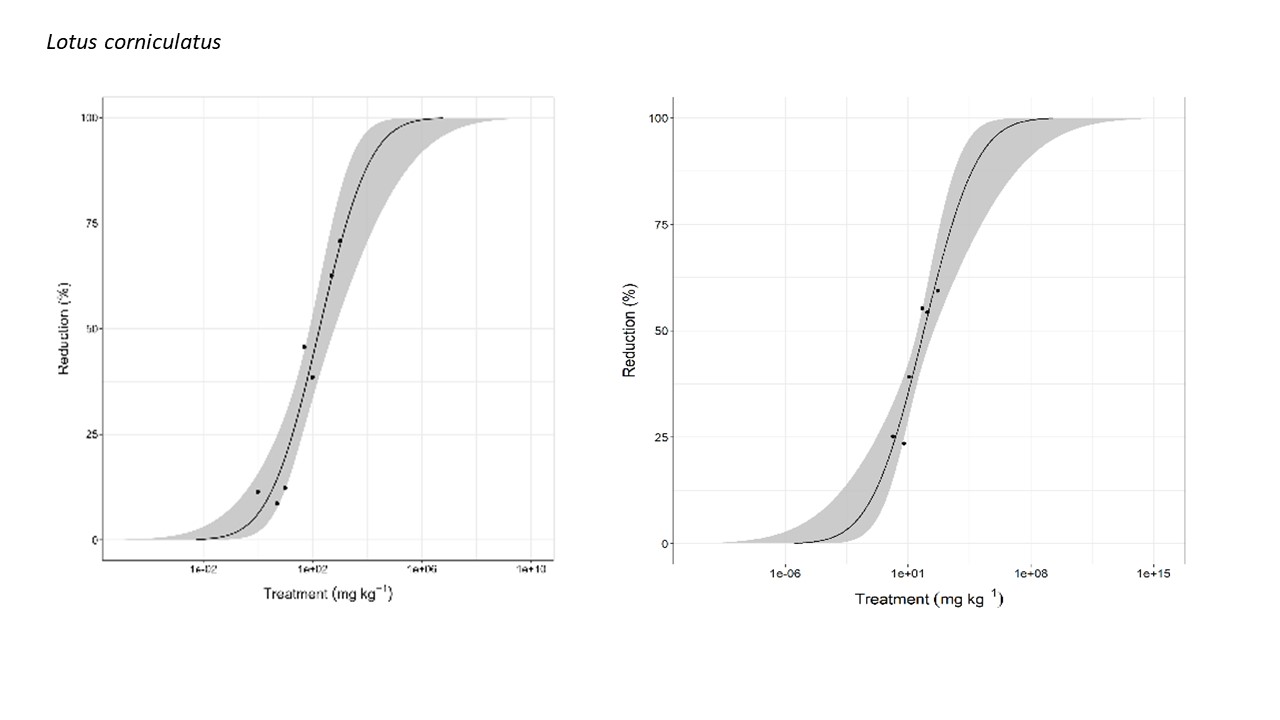

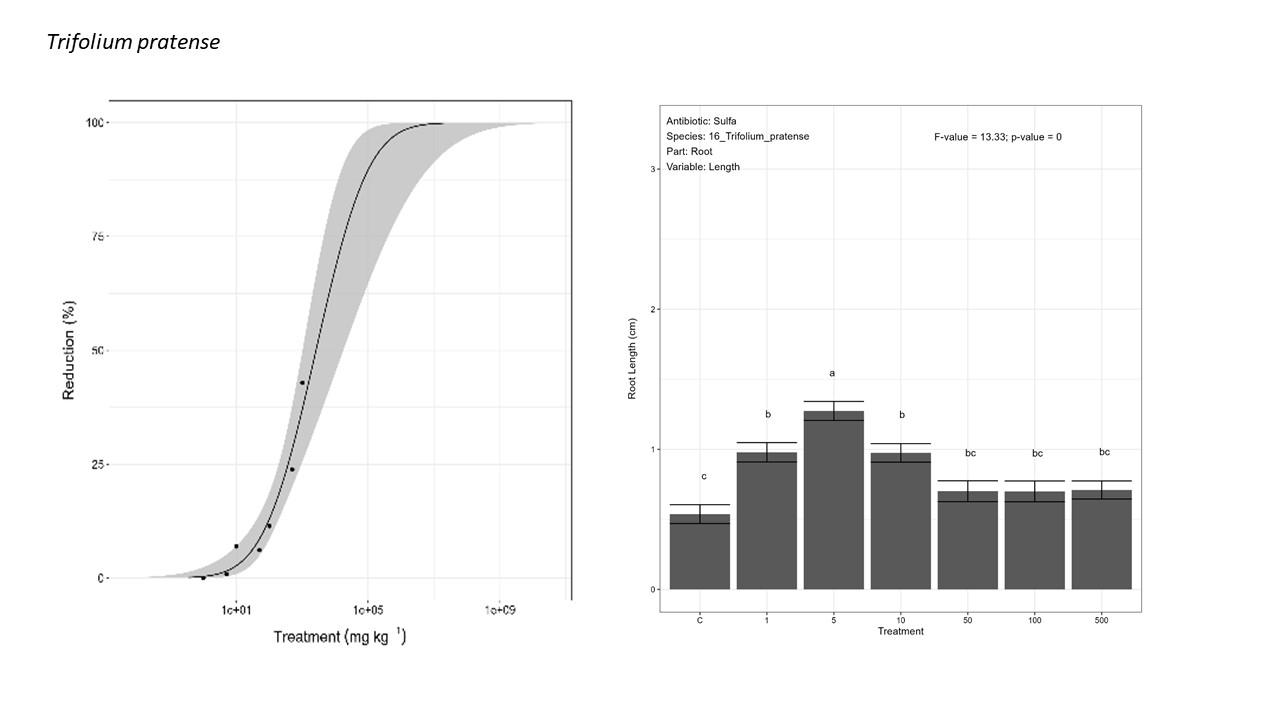

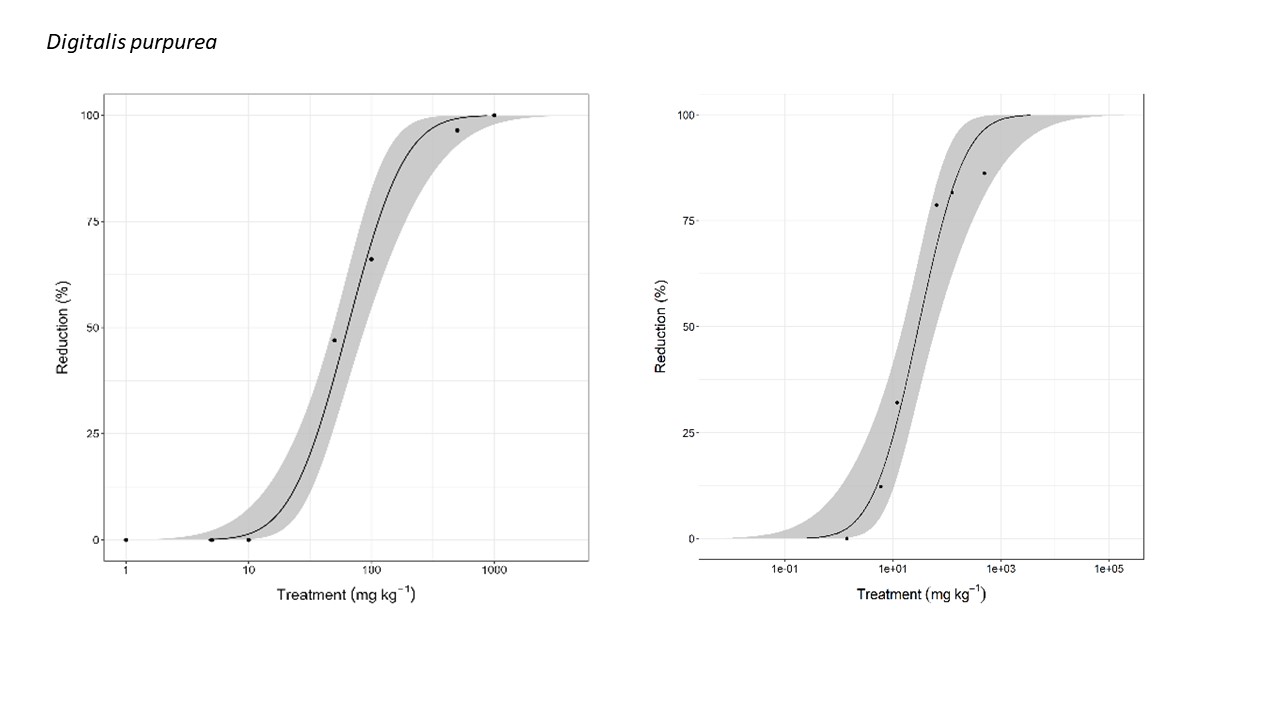

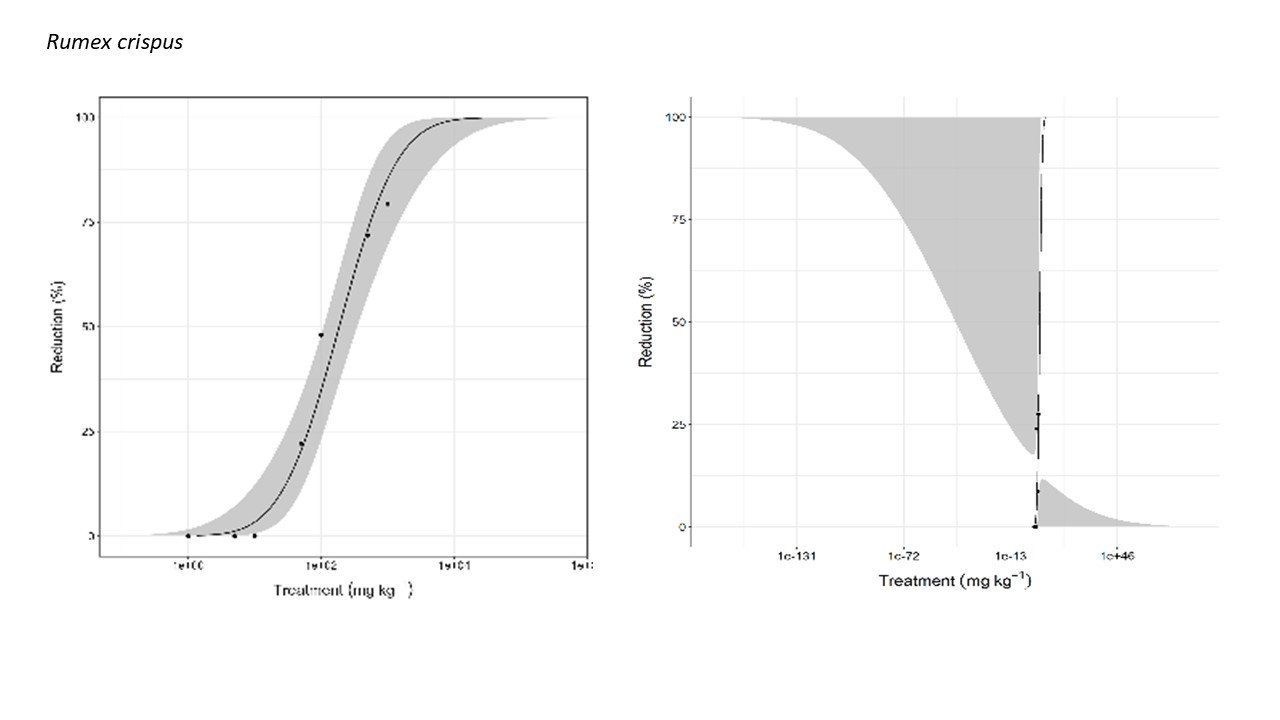

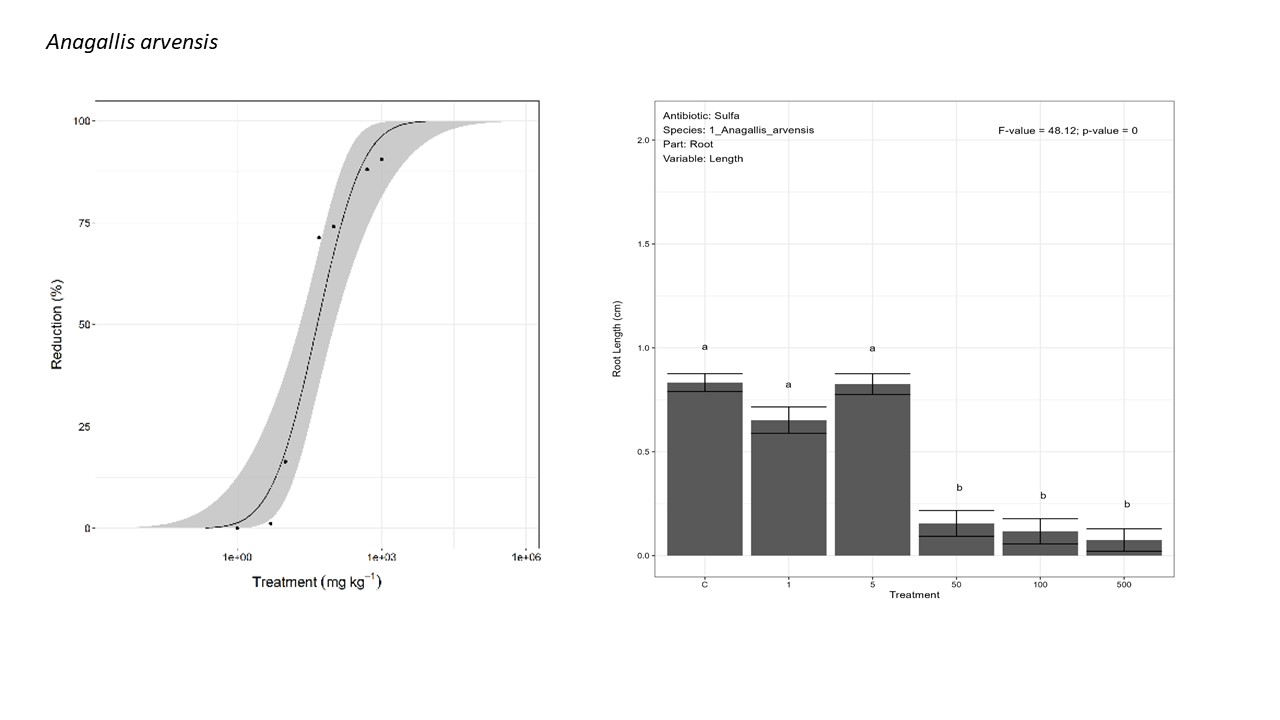

Supplement: Supplementary file 1 — Supplementary file1 (DOCX 1118 KB) [file 244_2024_1104_MOESM1_ESM.docx]
